# The Equilibria Between Monovalent Ions and Phosphatidylcholine Monolayer at the Air/Water Interface

**DOI:** 10.1007/s00232-013-9558-3

**Published:** 2013-05-11

**Authors:** Aneta D. Petelska, Zbigniew A. Figaszewski

**Affiliations:** 1 Institute of Chemistry, University in Bialystok, Al. J. Pilsudskiego 11/4, 15-443 Bialystok, Poland; 2 Laboratory of Electrochemical Power Sources, Faculty of Chemistry, University of Warsaw, Pasteur St. 1, 02-093 Warsaw, Poland

**Keywords:** Phosphatidylcholine, Monovalent ion, Complex formation equilibria, Monolayer, Langmuir trough

## Abstract

The effect of monovalent ion (Li^+^, Na^+^, Cs^+^) interaction with monolayers of phosphatidylcholine (lecithin, PC) was investigated at the air/water interface. We present surface tension measurements of lipid monolayers obtained using a Langmuir method as a function of monovalent ion concentration. Measurements were carried out at 22 °C using a Teflon trough and a Nima 9000 tensiometer. Interactions between lecithin and monovalent ions result in significant deviations from the additivity rule. An equilibrium theory to describe the behavior of monolayer components at the air/water interface was developed in order to obtain the stability constants and area occupied by one molecule of PC–monovalent ion complexes (PC^−^Me^+^).

## Introduction

Langmuir monolayers provide a unique way of studying two-dimensional (2D) materials at air/water interfaces, and investigations of Langmuir monolayers have uncovered a rich variety of physicochemical phenomena (Shapovalov 1998; Lee 2008; Fragneto et al. 2013; Petelska and Figaszewski 2013a). Langmuir monolayers are excellent model systems for membrane biophysics since a biological membrane can be considered as two weakly coupled monolayers. Langmuir monolayers are used for studies of chemical and biological interactions and reactions (Kaganer et al. 1999).

It is well known that the behavior of charged Langmuir monolayers is strongly affected by the ionic composition of the subphase solution. By now the influence of various inorganic monovalent and divalent ions (Binder and Zschörnig 2002; Sovago et al. 2007; Petelska and Figaszewski 2011, 2013b) as well as ionic organic dyes (Ahuja et al. 1993; Gregory et al. 1994) on the properties of some Langmuir monolayers has been widely investigated.

Ions destroy the natural hydrogen-bonded network of water, having effects similar to increased temperature or pressure (Leberman and Soper 1995). Small metal cations are strongly hydrated, with small or negative entropies of hydration, creating local orientation order and higher local water density (Binder and Zschörnig 2002). Ions that hydrate strongly are expected to compete with the lipid headgroups for the water and make water molecules less available for the headgroups. Moreover, complex formation of metal ions with the phosphate groups may be responsible for the increase in surface hydrophobicity of the membrane. These tendencies would reduce the range of the repulsive hydration force and, thus, promote fusion (Binder and Zschörnig 2002). It has been concluded from the swelling behavior of multilamellar phosphatidylcholine (PC) vesicles that alkali halides decrease the range of the hydration force following the Hofmeister series Li^+^, Na^+^, K^+^ (Korreman and Posselt 2001).

Binder and Zschörnig (2002) postulated that the biological effect of metal cations on phospholipid membranes depends on two events, namely, accumulation near the membrane surface and specific interactions with the headgroups. Membranes of anionic lipids are typically more strongly affected by metal cations than membranes of zwitterionic lipids because of stronger attractive coulombic forces. Data presented by Binder and Zschörnig (2002) illustrated the specific effect of ions on lipid properties. This high degree of specificity is obviously weakly related to the net charge of the lipid.

This work continues the systematic study of the ion effect of PC monolayers realized by Petelska and Figaszewski (2011, 2013b). In this article we present evidence for the formation of PC^−^Me^+^ (Me^+^ = Li^+^, Na^+^, Cs^+^) complexes at the air/water interface and calculate their stability constants. Knowledge of stability constants of the PC–monovalent ion system allows us to understand the processes that take place both in the monolayer itself and on its surface. The results can be used in quantitative descriptions of physical and chemical properties of biological membranes.

## Theory

During the formation of a mixed two-component monolayer (PC^−^Me^+^ ion) on a free electrolyte surface, the individual components (denoted by PC^−^ and Me^+^) can form complex (PC^−^Me^+^). Experiments were carried out at pH 7. At this pH the PC^−^ form is dominant, which was presented in previous work (Petelska and Figaszewski 2002, 2003). Therefore, in the present study we considered only the equilibrium between the anionic form of lecithin (PC^−^) and monovalent (Me^+^) ion.

The equilibria of such a system are described by the complexation reaction. Let us assume that a 1:1 complex is formed in a mixed monolayer at the air/water interface. The reactions (Petelska and Figaszewski 2011)

1 $$ {\text{PC}}^{ - } {\text{ + Me}}^{ + } \Leftrightarrow {\text{PC}}^{ - } {\text{Me}}^{ + } $$

may be described by the system of equations:

2 $$ K = \frac{{a_{{{\text{PC}}^{ - } {\text{Me}}^{ + } }} }}{{a_{{{\text{PC}}^{ - } }} \cdot a_{{{\text{Me}}^{ + } }} }} $$

3 $$ a_{{{\text{PC}}^{ - } }} A_{{{\text{PC}}^{ - } }} + a_{{{\text{PC}}^{ - } {\text{Me}}^{ + } }} A_{{{\text{PC}}^{ - } {\text{Me}}^{ + } }} = 1 $$

4 $$ a_{{{\text{PC}}^{ - } }} + a_{{{\text{PC}}^{ - } {\text{Me}}^{ + } }} = c_{{{\text{PC}}^{ - } }} $$

where $a_{{{\text{PC}}^{ - } }} ,a_{{{\text{PC}}^{ - } {\text{Me}}^{ + } }}$ (mol m^−2^) are the surface concentrations of components PC^–^, PC^–^Me^+^; $A_{{{\text{PC}}^{ - } }} ,A_{{{\text{PC}}^{ - } {\text{Me}}^{ + } }}$ (m^2^ mol^−1^) are the surface areas occupied by 1 mole of components PC^–^, PC^–^Me^+^; $a_{{{\text{Me}}^{ + } }}$ (mol dm^−3^) is the concentration of Me^+^ ions; $K_{{{\text{PC}}^{ - } {\text{Me}}^{ + } }}$ (dm^3^ mol^−1^) is the stability constant of the PC^–^Me^+^ complex; and $c_{{{\text{PC}}^{ - } }}$ (mol m^−2^) is the total surface concentration of lipid.

Elimination of $a_{{{\text{PC}}^{ - } {\text{Me}}^{ + } }}$ and $a_{{{\text{PC}}^{ - } }}$ parameters from the set of Eqs. 2–4 yields the basic equation

5 $$ y = m_{1} x_{1} + m_{2} x_{ 2} $$

where $y = 1 - c_{{{\text{PC}}^{ - } }} A_{{{\text{PC}}^{ - } }}$_,_ $m_{1} = K_{{{\text{PC}}^{ - } {\text{Me}}^{ + } }} A_{{{\text{PC}}^{ - } {\text{Me}}^{ + } }}$_,_ $x_{1} = c_{{{\text{PC}}^{ - } }} a_{{{\text{Me}}^{ + } }}$_,_ $m_{2} = - K_{{{\text{PC}}^{ - } {\text{Me}}^{ + } }}$_and_ $x_{2} = a_{{{\text{Me}}^{ + } }}$_._ $K_{{{\text{PC}}^{ - } {\text{Me}}^{ + } }}$ and $A_{{{\text{PC}}^{ - } {\text{Me}}^{ + } }}$ were calculated from the equations presented below:

6 $$ K_{{{\text{PC}}^{ - } {\text{Me}}^{ + } }} = - m_{2} $$

7 $$ A_{{{\text{PC}}^{ - } {\text{Me}}^{ + } }} = \frac{{m_{1} }}{{ - m_{2} }} $$

The parameters describing the complexes may be used to calculate theoretical points using the equation presented below (agreement between the theoretical and experimental values implies that the system is well described by the above equations):

8 $$ c_{{{\text{PC}}^{ - } }} = \frac{{K_{{{\text{PC}}^{ - } {\text{Me}}^{ + } }} a_{{{\text{Me}}^{ + } }} }}{{K_{{{\text{PC}}^{ - } {\text{Me}}^{ + } }} A_{{{\text{PC}}^{ - } {\text{Me}}^{ + } }} a_{{{\text{Me}}^{ + } }} + A_{{{\text{PC}}^{ - } }} - 1}} $$

## Materials and Methods

### Materials

3-sn-PC (99 %) from Fluka (Milwaukee, WI) was used in the experiment; it had been isolated from hen egg yolk. The 1-chloropropane solvent (>98 % pure) was supplied by Sigma-Aldrich (St. Louis, MO). Solutions were prepared by dissolving appropriate amounts of each material in 1-chloropropane at a concentration of 1 mg cm^−3^ and stored at 4 °C.

Electrolyte solutions (pH ~7) were prepared from triple-distilled water and lithium chloride (LiCl, 99 %), sodium chloride (NaCl, 99 %) and cesium chloride (CsCl, 99.9 %) from Sigma-Aldrich. Electrolyte solutions—0.5, 0.05, 0.005, 0.0025, 0.0005, 0.00025 and 0.00005 M—were used for experiments.

The water used in the experiments was prepared by triple distillation (the second distillation was performed over KMnO_4_ [POCh, Gliwice, Poland] and KOH [POCh] to remove organic impurities).

### Methods

The homemade, computer-controlled apparatus used for surface tension measurements was presented previously (Petelska and Figaszewski 2009).

Surface tension measurements were carried out at the air/water interface at 22 °C and expressed as surface pressure–area per molecule (*π*–*A*) isotherms. The measurement method was described previously (Petelska and Figaszewski 2009).

For all experiments, the trough was filled with monovalent ions (Me^+^) in triple-distilled water as the subphase. Monolayers were prepared by spreading a defined volume of a lecithin solution in 1-chloropropane on the aqueous subphase using a Hamilton microsyringe. 10 min were allowed for solvent evaporation and monolayer equilibration before an experiment was begun. The monolayer was continuously compressed to obtain the *π*–*A* isotherms using the glass barrier moved at a velocity of 0.03 cm s^−1^.

The Nima ST9002 computer program (Attension, Espoo, Finland) was used to calculate the surface pressure of the monolayer π as a function of the surface area per molecule *A*: *π* = *γ*–*γ*_0_ *=* *f*(*A*), where *γ*_0_ is the surface tension of the lipid-covered surface and *γ* is the surface tension of the bare air/water interface.

The system was enclosed in an acrylic box to minimize water evaporation, to ensure high humidity and to avoid contamination.

All of the reported values are highly reproducible and represent the average of at least five experiments. The standard deviation for surface area measurements was <1 %.

## Results and Discussion

In this article, we present surface tension measurements of lecithin monolayers obtained using a Langmuir method as a function of monovalent ion concentration. In this section we present evidence for the formation of PC–monovalent ion complexes (PC^–^Me^+^, where Me^+^ = Li^+^, Na^+^, Cs^+^) at the air/water interface and develop a system of equations to describe the complex formation. Using these equations, the stability constants of the PC^–^Me^+^ complexes were calculated.

### PC–Me^+^ Ion System, Where Me^+^ = Li^+^, Na^+^, Cs^+^

**Fig. 1:**
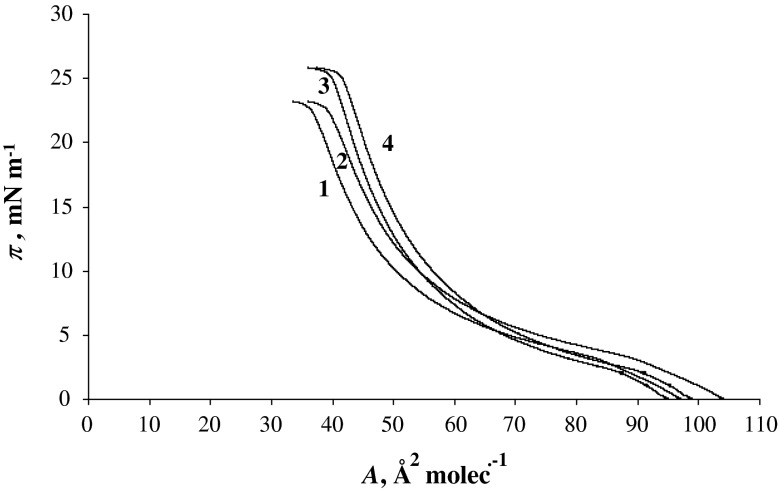
Surface pressure (*π*) versus surface area per molecule (*A*) isotherm of phosphatidylcholine monolayers: in pure water (*1*), 2.5 × 10^−4^ M Li^+^ ion concentration (*2*), 2.5 × 10^−4^ M Na^+^ ion concentration (*3*) and 2.5 × 10^−4^ M Cs^+^ ion concentration (*4*) as a subphase

Figure 1 presents four *π*–*A* isotherms of PC with absence of monovalent (Me^+^) ions (denoted by 1), PC with presence 2.5 × 10^−4^ M Li^+^ ions (denoted by 2), 2.5 × 10^−4^ M Na^+^ ions (denoted by 3) and 2.5 × 10^−4^ M Cs^+^ ions (denoted by 4). The PC isotherm is in satisfactory agreement with that previously reported (Brzozowska and Figaszewski 2002; Petelska and Figaszewski 2013a, b). The surface area for the PC molecule in pure water is 56 Å^2^. This value agrees with values reported in the literature (Brzozowska and Figaszewski 2002; Petelska and Figaszewski 2013a, b). The surface areas for the lecithin molecule in the presence of 2.5 × 10^−4^ M Li^+^, Na^+^ and Cs^+^ ion concentrations are 59, 60 and 65 Å^2^, respectively, which are presented in Fig. 1 (lines denoted by 2, 3 and 4).

In water, the phase behavior of PC has been well documented (Brzozowska and Figaszewski 2002; Aroti et al. 2004, 2007; Sovago et al. 2007; Petelska and Figaszewski 2013a, b). With decreasing surface area, the following regions have been identified: a gas phase, a liquid-expanded (LE) phase, a plateau characteristic of the coexistence of the LE and the liquid-condensed (LC) phase and the pure LC phase. With sodium ions present in the subphase, the compression isotherm for PC shifts to slightly higher surface pressures in the LE and LE + LC regions. The same effect has been observed previously (Aroti et al. 2004, 2007; Sovago et al. 2007) and was explained by disorder of the lipid chains induced by ions binding to the LE phase. At higher surface pressure, sodium has no effect on the compression isotherm, indicating no significant interaction of Na^+^ with the LC phase: the ions are probably being “squeezed out” from the headgroup region (Sovago et al. 2007).

**Fig. 2:**
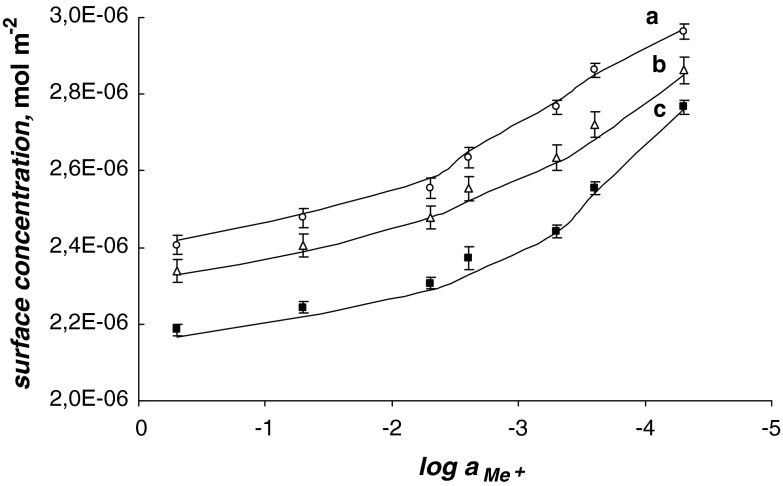
Dependence of total surface concentration of phosphatidylcholine, $c_{\text{PC}-}$, on the logarithm of monovalent ion concentration, $a_{{{\text{Me}}^{ + } }}$, where Me^+ ^= Cs^+^ (*a*), Na^+^ (*b*), Li^+^ (*c*) (*Filled square* experimental values, *Solid line* theoretical values) at a surface pressure of ~25 mN m^−1^

The total surface concentrations of PC versus the logarithm of monovalent Me^+^ ion concentration, where Me^+^ = Li^+^ (a), Na^+^ (b), Cs^+^ (c), is depicted in Fig. 2. In Fig. 2, the experimental points are compared with the values calculated using Eq. 8 (depicted as a line). Figure 2 refers to the above description (see “Theory”) where the distribution of the monolayer components on the air/water interface of the lipid layer has been assumed to be uniform. As seen in Eq. 4, the total surface concentration of the lecithin membrane is a sum of the surface concentration of its components, i.e., PC^–^ and PC^–^Me^+^.

According to Eqs. 6 and 7, it is possible to determine the area occupied by one molecule of the PC^–^Me^+^ complex and the stability constants of these complexes, which are presented in Table 1. $K_{{{\text{PC}}^{ - } {\text{Me}}^{ + } }}$ values, where Me^+ ^= Li^+^, Na^+^, Cs^+^, were calculated by inserting the experimental data into Eq. 6 and were relatively high, which provides additional evidence for the prevalence of a 1:1 complex in PC monolayer with the presence of monovalent ions.

**Table 1:** Physicochemical parameters for 1:1 of phosphatidylcholine–monovalent ion complexes

Examined system	Surface area occupied by one molecule of complex (Ǻ^2 ^mol^−1^)	Stability constant of examined complex (m^2^ mol^−1^)	Complex formation energy (Gibbs free energy) (kJ mol^−1^)
PC^–^Li^+^	57 ± 0.57	1.80 × 10^2^	−12.85 ± 0.64
PC^–^Na^+^	59 ± 0.59	2.50 × 10^2^	−13.66 ± 0.68
PC^–^K^+^ (Petelska and Figaszewski 2011)	60 ± 0.60	3.26 × 10^2^	−14.18 ± 0.71
PC^–^Cs^+^	62 ± 0.62	7.58 × 10^2^	–16.41 ± 0.82

Knowing the area occupied by the PC^–^Me^+^ complex and the stability constant value of this complex, the theoretical values of the surface concentration of the lecithin monolayer in the presence of Me^+^ ions were calculated using Eq. 8. The theoretical values obtained are presented in Fig. 2 and are marked by lines; points on the same figure show the experimental values. It can be seen that the agreement between experimental and theoretical points is very good, which verifies the assumption of the formation of the PC^–^Me^+^ complexes in the lipid monolayer. Good agreement between the experimental and the theoretical points verifies the assumption of a 1:1 complex in the PC monolayer. The lack of variation between theoretical and experimental points indicates that the theoretical model (see “Theory,” above) is sufficient to describe the interaction in the lecithin–monovalent ion system. The agreement between the experimental results and the model predictions for the PC^–^Me^+^ ion system justifies the statement that other complexes do not represent a significant component of this system.

In our opinion the monovalent ion Me^+^ interacts with the headgroup moiety, most likely the phosphate group. The Me^+^ ion and phosphate are known to form a strong ion pair in water, and the strength of this interaction is likely to be increased in the lipid headgroup region where the dielectric permittivity of the surrounding (and thus the electrostatic screening of charges) is reduced (Zhang et al. 1991).

The results presented previously (Kotynska and Figaszewski 2005; Sovago et al. 2007) indicate that monovalent ions, like sodium, have very little effect on lipid monolayers and bilayers. Association constants of the surface groups with the solution ions were presented previously (Kotynska and Figaszewski 2005). The *K*_BOH_, *K*_BCl_, *K*_AH_ and *K*_ANa_ constants are equal to 5.35 × 10^9^, 0.218, 5.58 × 10^5^ and 0.051 (m^3^ mol^−1^), respectively.

Table 1 summarizes the physicochemical parameters for a monolayer composed of lecithin and four monovalent ions: Li^+^, Na^+^, K^+^ (Petelska and Figaszewski 2011) and Cs^+^.

As can be seen from the results, the areas occupied by complexes being formed between lecithin and the monovalent ion (Li^+^, Na^+^, K^+^ and Cs^+^) increase along with the increase of the radius of the ion. Along with the increase of the radius of the ion also the stability constant of complexes being formed between the PC and the monovalent ion (Li^+^, Na^+^, K^+^ and Cs^+^) increase.

The increase of stability constant values of complexes being formed between the lecithin and the monovalent ion (Li^+^, Na^+^, K^+^ and Cs^+^) along with the increase of the radius of the ion can be caused by their being found in an aquatic environment of hydration layers, on which the size and the permanence depend: from the structure of the dissolved substance, pH of the solution, temperature and the connections between different components present in the environment. Total hydration layers, e.g., 16 water molecules, contain the ion of sodium but not the of ion of potassium (−10).

Comparing the examined connections typical of the value of appointed areas in the above table implies that the appointed area falling to one molecule of the PC–monovalent ion (Li^+^, Na^+^, K^+^ and Cs^+^) complex takes out appropriately 57, 59, 60 and 62 Å^2^ mol^−1^ and is bigger than the area occupied by a single molecule of lecithin (56 Å^2^ mol^−1^). It can suggest that the joining monovalent ion causes a parting of lecithin molecules in the monolayer. It is possible to estimate that the difference between the value of the area occupied by the lecithin–monovalent ion complex and the area occupied by the pure lecithin takes out 3–8 Å^2 ^mol^−1^. It is probably connected with the area occupied by the monovalent ion. The radius of the Na^+^ ion amounts to about 1.0 Å; however, Li^+^ amounts to about 0.7 Å (Colton et al. 1995).

## Conclusions

The following conclusions can be drawn on the grounds of the parameters describing the studied complexes.

1. The stability constant of the PC^–^Li^+^ complex is 1.80 × 10^2^ m^2^ mol^−1^, whereas the stability constants of the other complexes are 2.50 × 10^2 ^m^2^ mol^−1^ in the case of the PC^–^Na^+^ complex and 7.58 × 10^2^ m^2^ mol^−1^ in the case of the PC^–^Cs^+^ complex.
2. The experimental area occupied by one PC^–^Li^+^ is 57 Ǻ^2^ mol^−1^, whereas the areas occupied by other complexes are 59 Ǻ^2^ mol^−1^ for PC^–^Na^+^ and 62 Ǻ^2 ^mol^−1^ for PC^–^Cs^+^.
3. Good agreement of the experimental and theoretical points verifies the assumption of formation a 1:1 complex in the lipid monolayer. Lack of variance between points indicates that complexes at different stoichiometries or associates are not possible in the PC–monovalent ion membranes.
